# Sensitivity of Inter-Point Distance Measurement to Control-Point Coordinate Error in a Panoramic Camera

**DOI:** 10.3390/s26196300

**Published:** 2026-10-05

**Authors:** Rihards Krišlauks, Rainers Kozlovskis, Teodors Eglītis, Janis Judvaitis

**Affiliations:** 1 Latvijas Mobilais Telefons SIA, LV-1039 Riga, Latvia; teodors.eglitis@lmt.lv; 2 Institute of Electronics and Computer Science, LV-1006 Riga, Latvia

**Keywords:** camera calibration, control-point coordinate error, ground-plane homography, inter-point distance measurement, Monte Carlo methods, sensitivity analysis, stitched panoramic camera, traffic monitoring

## Abstract

Vision-based traffic monitoring recovers metric quantities such as inter-vehicle distances by mapping the image to the ground plane through a planar homography. In deployed systems, this homography is often fitted from ground control points whose world coordinates are obtained from satellite orthoimagery, where each coordinate is uncertain by about one image pixel. The impact of this control-point coordinate error on the measured distance has not been quantified for a single fixed camera. This paper evaluates a 180-degree panoramic camera (AXIS P3807-PVE) on two adjacent ground-plane regions, each referenced to RTK-surveyed control points and mapped by a separate homography. Control-point coordinates are perturbed with isotropic Gaussian noise, the homography is refitted, and the resulting change in inter-point distance between held-out evaluation points is measured against the unperturbed fit. The distance error is dominated by random scatter rather than systematic bias, and the scatter grows approximately linearly with the coordinate noise, at about 0.7 to 0.8 times the per-coordinate standard deviation within the tested range. The results indicate that distance error can be predicted from control-point coordinate accuracy, and that relative precision of those coordinates matters more than absolute georeferencing.

## 1. Introduction

Vision-based traffic monitoring increasingly relies on recovering metric information (vehicle positions, inter-vehicle distances, and speeds) from camera imagery rather than from dedicated sensors. When the scene of interest lies on the road surface, these quantities are obtained by mapping image coordinates to the ground plane through a planar homography [1,2]. Many such installations use a single wide-field-of-view camera, deployed and used as-is (for example, [3]), and the geometric accuracy of the resulting ground-plane measurements directly determines the quality of the quantities these systems produce.

The calibration that underpins this mapping has been extensively automated, typically by inferring the camera geometry from scene cues such as vehicle motion and vanishing points [4,5]. In deployment, however, an alternative is often preferred: the homography is fitted directly from ground control points whose world coordinates are surveyed, for example, using satellite positioning. These world coordinates are not exact. When read from satellite imagery, a control point’s coordinate is limited by the image resolution, of the order of one pixel (10 to 25 cm in local Latvian orthophoto maps [6]; we assume roughly 15 cm on the ground for this article). Deployed accuracy is usually reported as a single figure obtained under favourable conditions, which does not show how sensitive it is to this control-point coordinate error. The question matters most for cameras used as-is, where a single homography must also absorb whatever residual geometric distortion the deployed image carries.

Prior work establishes that automatic calibration attains a relative-distance accuracy of the order of a few percent [4,7], and that the localisation of control points is the dominant factor limiting homography accuracy [8]. In aerial photogrammetry, the effect of control-point coordinate accuracy on mapping accuracy is well studied [9,10], but that setting uses many overlapping images and bundle adjustment, so its geometry differs from a single fixed camera. For a deployed traffic camera that fits one planar homography from control points, with no overlapping views, the effect of decimetre-level error in the control-point world coordinates on the measured inter-point distance has not been quantified. This paper asks how much that error changes the distance.

We study a single deployed camera: a 180° panoramic camera (AXIS P3807-PVE) [11] that combines four sensors, each with its own lens, into one seamlessly stitched 180° image [12]. We use the two sensors that cover the left half of the field of view, which gives two adjacent ground-plane regions (slices), each on the road surface next to the camera. The imagery is used as-is, as delivered by the camera, and each slice is mapped to metric ground-plane coordinates by its own planar homography fitted from RTK-surveyed control points. The control points define a local metric frame, so only relative inter-point distances are evaluated and absolute georeferencing is not needed. Our main contribution is a Monte Carlo characterisation of how error in the world coordinates of the control points propagates into the measured inter-point distance, reported as signed bias and scatter against the unperturbed homography. We do not propose a new calibration method and apply no correction beyond what the camera already performs; the aim is to characterise an as-deployed measurement pipeline, not to optimise it.

### Related Work

In computer vision and photogrammetry, metric quantities can be recovered from a single view of a planar surface using projective geometry [1]. Refs. [2,13] formalised measurement on and between planes from uncalibrated imagery and analysed how localisation uncertainty propagates into the resulting measurements. For ground-based traffic monitoring, this reduces to a planar homography mapping image coordinates to metric ground-plane coordinates, from which inter-point distances, object positions, and speeds are obtained. The accuracy of any such measurement is therefore governed by the quality of the estimated homography and of the point correspondences from which it is computed.

**Calibration of traffic-surveillance cameras.** Because chessboard-style intrinsic calibration [14] is impractical for in situ surveillance installations, the dominant line of work estimates the camera geometry directly from the traffic scene. Refs. [4,15] recover two vanishing points from vehicle motion and edge orientation to obtain the camera intrinsics and the road-plane orientation without prior knowledge, and [5] adds automatic scene-scale inference by aligning rendered 3D vehicle models. Later methods detect vanishing points with convolutional networks [16,17], calibrate from landmarks on rigid objects [18,19], or target large-scale automation across camera networks [20]. These approaches infer scale from scene cues; an alternative, used by deployed measurement systems and adopted here, is to fit the homography directly from surveyed ground control points, trading scene assumptions for a dependence on the accuracy with which those control points are placed in the image.

**Datasets and accuracy benchmarks.** Comparable evaluation of such systems was enabled by the BrnoCompSpeed dataset [7], which provides hour-long sequences with LiDAR-verified speeds and road markers at known distances. Because monocular calibration recovers geometry only up to scale, accuracy is conventionally reported through scale-invariant relative quantities, such as ratios of measured-to-true inter-point distances [7,16]; we adopt the same relative-distance formulation. State-of-the-art automatic calibration reports a relative-distance accuracy of around two percent [4], with further reductions in relative-distance error from improved vanishing-point detection and scale inference [5], which sets the accuracy scale against which deployed ground-plane measurement should be interpreted. Vision-based speed measurement, which relies on the same image-to-road mapping, is surveyed in [21], and assessments of video-extracted NGSIM trajectories found errors large enough to distort inter-vehicle spacing [22,23]. In the CityFlow benchmark [24], traffic-camera homographies were fitted from points read from Google Maps, with an average reprojection error of 11.52 pixels attributed to the limited precision of the map.

**Control-point accuracy and homography sensitivity.** The accuracy of a homography estimated from point correspondences is limited primarily by the localisation of those points. Ref. [8] shows that the spatial configuration of n≥4 control points strongly affects both the conditioning of the direct linear transform and the resulting homography and pose accuracy, and that point-localisation error is decisive; normalisation of the correspondences is the standard remedy for the underlying numerical sensitivity [25]. These analyses are, however, largely confined to simulation or to the geometric arrangement of the points, in application contexts that differ from georeferenced ground-plane measurement. The sensitivity of measured ground distances to decimetre-level error in the world coordinates of surveyed control points has, to the authors’ knowledge, not been quantified for a deployed single-homography camera.

**Homography validation against independent references.** The validation of homography-based measurement against independent references has been addressed most directly in structural health monitoring. Ref. [26] evaluates UAV-based homography for displacement monitoring across controlled, laboratory, and full-scale bridge tests with direct comparison to LVDT and total-station references, and reports that accuracy is contingent on appropriate reference-point configuration and imaging geometry. That setting operates at a sub-millimetre-to-millimetre scale, and measures out-of-plane displacement of a tracked target.

**Positioning of this work.** Thus, automatic calibration of traffic-surveillance cameras is mature, and the relative-distance evaluation protocol and its few-percent accuracy scale are well established. In aerial photogrammetry, the effect of control-point coordinate accuracy and distribution on mapping accuracy has also been studied in detail, including with simulated coordinate noise and held-out check points [9,10]. That work uses many overlapping images and bundle adjustment, where redundancy across views changes how control-point error behaves. A single fixed camera that fits one planar homography has no such redundancy. A shift applied to all control points together is absorbed by the homography and does not change the relative distances between projected points; only the per-point scatter and a uniform scale error change those distances. We evaluate a deployed 180° camera, used as-is and referenced to RTK-surveyed control points, and quantify through Monte Carlo perturbation how decimetre-level error in the control-point world coordinates propagates into measured inter-point distance.

## 2. Materials and Methods

We do not propose a new method: the object of study is an existing, as-deployed pipeline, applied independently to each slice.

Evaluation uses a single 180° panoramic camera (AXIS P3807-PVE) [11], which combines four sensors, each with its own lens, into one seamlessly stitched 180° image on-device [12]. The on-device stitching projects the sensor images onto a common surface and corrects barrel distortion, so the delivered image is not a raw fisheye frame; we use it as delivered, without further correction. We use the two sensors that cover the left half of the field of view, which gives two adjacent regions on the road surface next to the camera; we call each region a slice. The studied area is the tarmac close to the camera (about 5 to 6 m wide); far parts of the field of view are not used. A separate ground-plane homography is estimated for each slice, and each measurement is evaluated within a single slice. This is necessary because the stitched 180° image is not a perspective image and cannot be mapped to the ground by a single homography, so a panoramic camera is used for ground-plane measurement one sensor region at a time. The two slices cover both sensor positions of the camera, an outer and an inner one; the right half of the view mirrors them and is therefore not studied separately. The two sensors have different viewing angles and distortion, so the two slices are two test conditions, not repeats of the same one. Target-based calibration [14], which also covers omnidirectional cameras and multi-camera systems, including those without overlapping views [27,28,29], could be applied to each slice and would reduce the residual distortion, but it does not remove the error studied here. The mapping from the image to the road must still be fitted from points of known world coordinates on the road, so the control points used here act as a sparse calibration target on the ground. Unlike the corners of a printed pattern, their world coordinates are not known by construction: here they are surveyed on site, and, where the road cannot be accessed, they are read from orthoimagery. It is the error in these world coordinates, not in locating the points in the image, that this study models.

**Figure 1:**
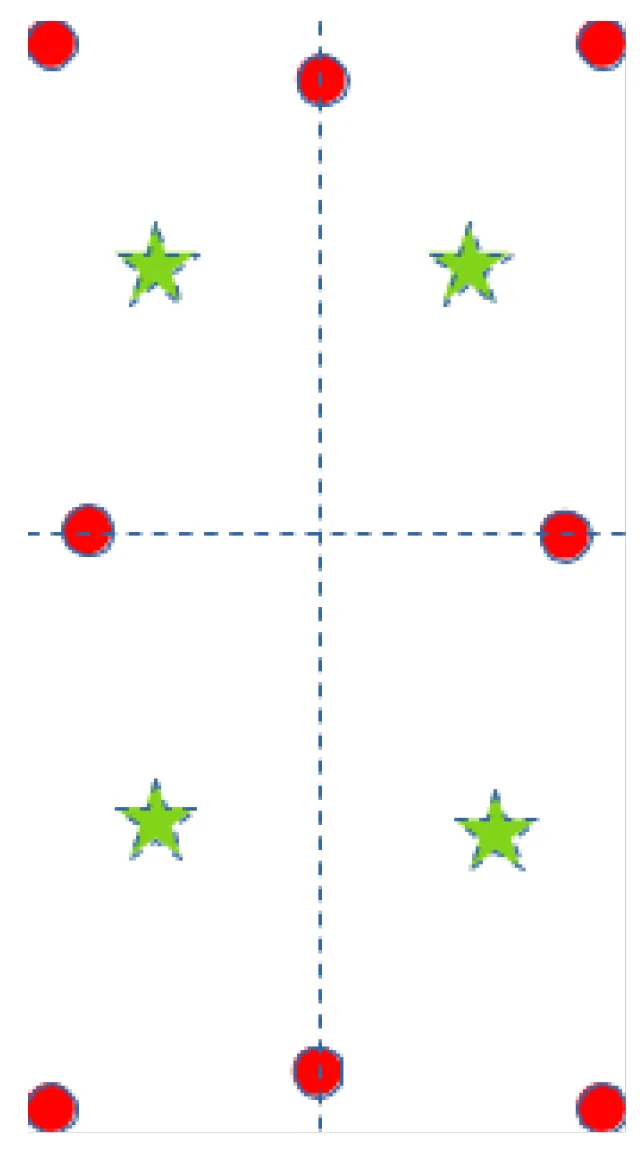
Control and evaluation point schema per slice. Red are control points, green are evaluation points, locations are approximate.

**Control points and homography.** For each slice, eight control points on the road surface are used to fit the homography: four at the corners of the usable area and four near the middle of its sides (Figure 1), with the side points offset inwards so that no three control points lie on a straight line (a near-collinear set makes the fit unstable). The corners bracket the area so that evaluation is by interpolation, not extrapolation. Each control point is a fixed physical marker lying flat on the road (height ≈0), and its position is measured by RTK GNSS. Coordinates are transformed to the LKS-92/Latvia TM projection (EPSG:3059); this is enough because only relative distances are used. The homography *H* that maps image coordinates to this metric frame is estimated per slice with the RANSAC robust estimator [1], and the per-point reprojection residual is reported as a fit diagnostic: a residual structure that grows across the slice indicates geometric distortion not absorbed by the planar model. In our measurements, the RTK horizontal standard deviation is about 1.5 cm (from the receiver error estimate), well below the coordinate error studied below, so the RTK points are treated as practical truth. The fix type and reported standard deviation are logged for each point.

**Evaluation points and distance metric.** Accuracy is evaluated on a separate set of held-out points, never on the control points. In each slice, we use four evaluation points, placed near the centre of each quadrant of the control-point area (Figure 1) and kept off the straight lines between control points; their positions are also measured by RTK. The reported quantity is the inter-point distance: for a given homography, the evaluation points are projected from the image to the metric frame, and the distances between all pairs are computed. All errors are reported in absolute form (m) and relative form (% of the true distance), where the true distance is taken from the RTK coordinates of the evaluation points.

**Baseline accuracy.** With the unperturbed homography, the projected inter-point distances are compared with the true distances. We report the signed mean (bias) and the standard deviation, in absolute and relative form. Because both the control points and the evaluation points are surveyed with the same RTK setup, this baseline figure reflects fit consistency rather than an independent accuracy measurement; it is reported as a diagnostic, not as the paper’s main result. Each slice is assumed planar; road non-planarity, if present, adds to this baseline error but not to the sensitivity result.

**Sensitivity to control-point coordinate error.** The main result is how error in the control-point world coordinates changes the measured distance. This models the error of reading a control point from satellite imagery, where the world coordinate is uncertain by about the satellite pixel size, of the order of 15 cm on the ground (typical of sub-metre orthoimagery, within the 10 to 25 cm reported for local Latvian orthophoto maps [6]). We add isotropic Gaussian noise $N(0,\sigma^{2})$ independently to the world coordinates (x,y) of each control point, re-solve the homography, project the held-out evaluation points, and recompute the pairwise distances. The displacement of each distance from its unperturbed value is the sensitivity; it is measured against the unperturbed homography and so does not depend on ground truth. We sweep σ∈{5,7.5,15,22.5,30} cm, which is about {1/3,1/2,1,3/2,2} satellite pixels at 15 cm per pixel, and use N= 10,000 draws per σ. For each σ, we report the signed mean (bias) and the standard deviation of the distance displacement, in absolute and relative form. The noise is added independently per point rather than as one shared shift because a shared shift (a global georeference offset of the orthoimage) is absorbed by the homography and cancels in relative distances, so only the independent per-point scatter, which does not cancel, and a small uniform scale error remain. The camera (image) side is held fixed by localising each point on a stable physical marker and is treated as a constant noise floor, not a variable.

**Error sources.** The reported error is a combined property of the as-deployed pipeline and comes from three main sources. First, error in the control-point world coordinates (satellite-clicking), the variable studied by the Monte Carlo sweep above. Second, model misspecification: a single planar homography applied to imagery that still carries residual projection distortion from the camera’s on-device stitching, seen as the baseline bias and the reprojection residual. Third, image-side localisation of the points, held fixed and treated as a noise floor.

**Full experiment workflow.** Following the previously described surveying procedure, RTK GPS measurements were collected at predefined sampling locations, with the process video-recorded for image-side localisation. The captured points were matched to image pixels and converted from WGS-84 to LKS-92. For camera segments 1 and 2, a base-case evaluation without noise was first conducted by estimating the homography matrix *H* using tX points. The fitted homography was used to reproject these points to obtain the control error, while the evaluation points eX were projected to obtain eX’ and evaluation bias. Subsequently, for each noise level σ, an independent offset $\left(\delta x,\delta y\right)∼N\left(0,\sigma^{2}I\right)$ was added to the world coordinates of the tX points, with their image coordinates unchanged, and *H* re-estimated. This process was repeated *N* times per noise level, with the resulting projections of eX’ used to calculate point scatter, distance bias, and standard deviation.

### Experimental Setup

**Site and camera.** Data were collected at the premises of the Institute of Electronics and Computer Science, 14 Dzerbenes Street, Riga, Latvia. The AXIS P3807-PVE camera was mounted at a height of 3 m on a pole at the roadside on private territory. The image resolution of each sensor is 912×2052 pixels. Data were collected under dry, daylight conditions, with no shadows obstructing the view.

**Figure 2:**
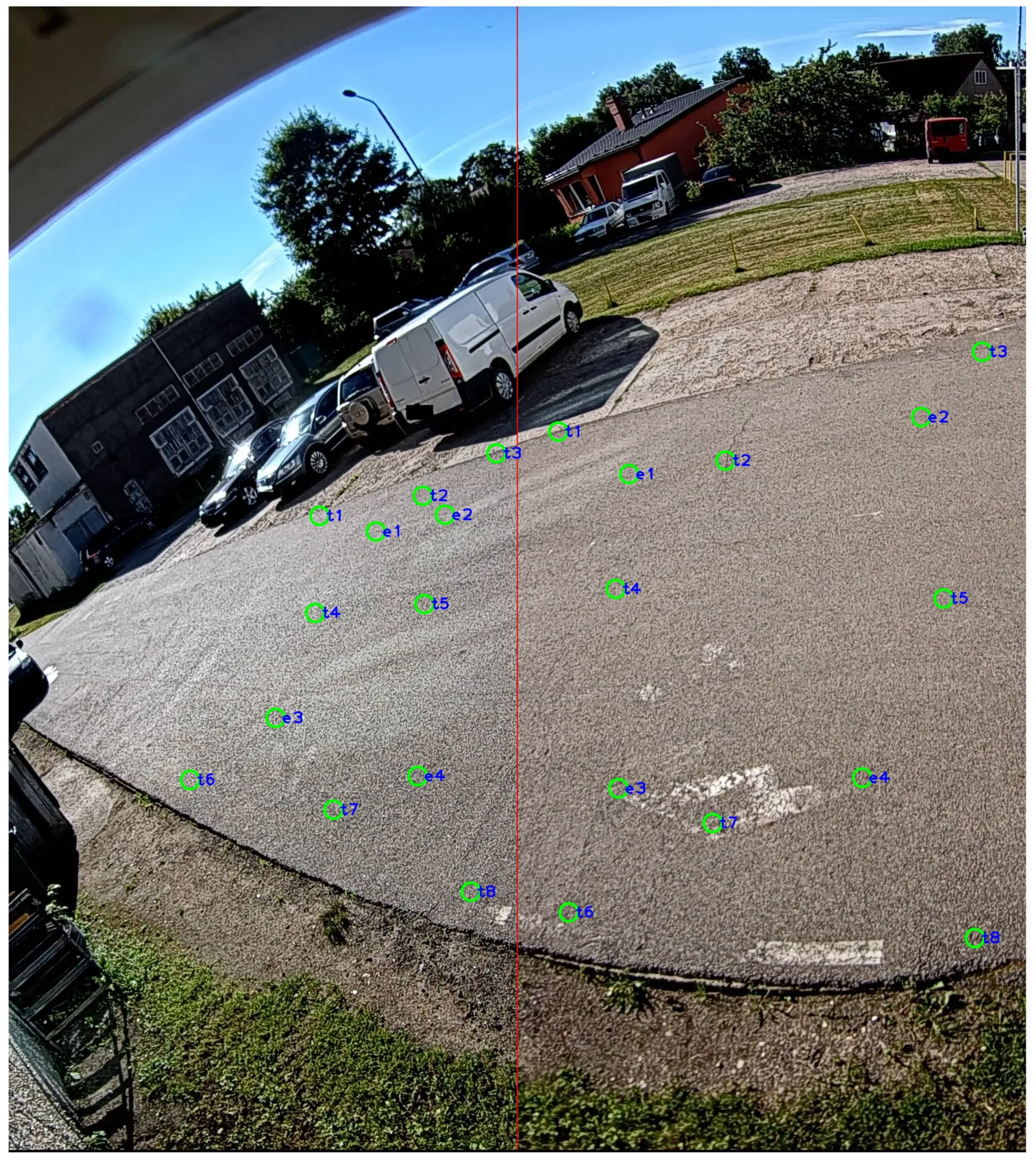
Control and evaluation points on both slices. The red line marks the slice border; slice 1 on the left and slice 2 on the right. Points tX are for control and eX are for evaluation. Precise sample position marked by the center red pixel.

**RTK equipment and point marking.** World coordinates were measured using two Emlid Reach M2 multi-band RTK GNSS modules with multi-band helical antennas: one for the survey points, the other as an RTK base station on the roof of an adjacent seven-storey building. Each point was occupied for at least 1 min before recording: an example of representative RTK horizontal precision for a single occupied point is given in Table 1. All control and evaluation points were surveyed in one session from one base station, so session-common errors largely cancel in the point-to-point differences. Control and evaluation points (Figure 2) were localised in the images using a fixed white antenna base employed with the RTK setup. The antenna base, measuring 10×4 cm with a height of 9 cm, was placed at each surveyed point, facing the camera, and remained clearly visible in the acquired video recordings. The pixel position corresponding to the road surface between the antenna legs was picked for each point sample, so the picked point lies on the ground plane at the same offset from the surveyed point throughout. At the near edge of the studied area, the 10 cm width of the antenna base spans about 30 pixels in slice 1 and 50 in slice 2, so one pixel is well under a centimetre on the ground there.

**Software and data availability.** The homography was estimated by findHomography (method RANSAC, with ransacReprojThreshold = 3 m, maxIters = 2000, and confidence = 0.995; the threshold is in the units of the destination points, which here are metric ground coordinates), and then used to project points with perspectiveTransform from the OpenCV (v4.13.0) library. The Monte Carlo perturbation and distance computation were implemented in Python 3.12 using the NumPy library. The gathered and used data, together with the source code, can be found in the Zenodo repository https://doi.org/10.5281/zenodo.22749074.

## 3. Results

Results are reported separately for the two slices, since they correspond to different sensors with different viewing angles and distortion. For each slice, we first report baseline accuracy against the unperturbed homography, then the sensitivity of measured distance to control-point coordinate error under Monte Carlo perturbation.

### 3.1. Baseline Accuracy: Slice 1

Fitting *H* from the eight control points and projecting the four evaluation points gives a per-point position residual (projected vs. RTK) with a magnitude of 3.9±2.1 cm at the control points and 4.3±2.1 cm at the evaluation points, and a control-point reprojection RMSE of 4.4 cm (mean ± std over points; Table 2). Both quantities are RTK-vs-RTK fit-consistency diagnostics, not an independent accuracy check; we report them as such.

The six pairwise distances between evaluation points are all shorter under the unperturbed homography than under RTK truth: the signed bias is −6.3±3.7 cm (mean ± std over the six pairs), or −1.70±0.52% in relative terms (mean across the six evaluation-point pairs; Table 3). The consistent sign across all pairs indicates a systematic underestimation of the evaluation-point distances.

The error is not consistent with a simple uniform scale factor. The absolute error increases with pair distance, from 1.3 cm at 142 cm to 11.7 cm at 522 cm, but this alone is expected under a scale error. The relative error, however, also increases over the same range, from 0.9% to 2.4%, whereas a uniform scale factor would produce an approximately constant relative error. This indicates that the error has both a distance-independent and a distance-dependent component.

### 3.2. Baseline Accuracy: Slice 2

The residuals are larger throughout for slice 2 (Table 4): 6.6±4.3 cm at the control points and 9.1±2.0 cm at the evaluation points, with a control-point reprojection RMSE of 7.9 cm, as expected given this sensor’s more oblique view and stronger distortion.

The evaluation-point distances are again all underestimated (Table 5), with a signed bias of −14.0±5.4 cm (−3.99±0.75%), about 2.3× the slice 1 underestimation; the sign is again consistent across all six pairs.

The same pattern holds as in slice 1: both the absolute error (from 3.2 cm at 130 cm to 19.0 cm at 425 cm) and the relative error (2.4% to 4.5%) grow with distance, so the error again has both a distance-independent and a distance-dependent component and is not a pure scale factor.

### 3.3. Sensitivity to Control-Point Coordinate Error: Slice 1

Perturbing the control-point world coordinates with isotropic Gaussian noise (N= 10,000 draws per σ) produces distance error dominated by random scatter rather than systematic bias. Across all noise levels, the mean distance displacement stays near 1 cm (Table 6, averaged across the six evaluation-point pairs), while the standard deviation of the displacement grows from 3.4 cm at σ=5 cm to 20.3 cm at σ=30 cm.

The scatter is linear in the control-point noise: the mean displacement standard deviation is a near-constant 0.67 times σ across the whole range. In relative terms, the mean scatter rises from 1.1% at σ=5 cm to 6.5% at σ=30 cm.

The underestimation seen in the unperturbed baseline does not reappear here: the perturbation bias is small and not consistently signed, suggesting that it originates from the nominal homography fit rather than from random control-point coordinate error.

Across the six evaluation-point pairs, longer-baseline pairs show larger absolute scatter, but it grows more slowly than distance itself (the longest pair, e1–e4 at 522 cm, has about 2.3× the scatter of the shortest, e1–e2 at 142 cm, against a 3.7× distance ratio). Relative scatter is therefore larger for short pairs: at σ=15 cm, it ranges from 2.4% for the longest pair to 4.7% for the shortest. This is the opposite of the baseline relative error, which grew with distance, and indicates that short distances are the more sensitive to control-point coordinate error.

The scatter is not uniform across the four evaluation points. Resolving the perturbation response per point (rather than per pair, which also scales with baseline length), the positional scatter (RMS over draws) is linear in σ for every point, with a point-specific slope that ranges from 0.71 for e1 to 0.89 for e4 (Table 7). The same control-point noise therefore produces about 25% more positional scatter at e4 than at e1. This sensitivity tracks distance from the camera: e1, imaging ground far away, is the least sensitive, while e4, imaging ground close by, is the most sensitive.

### 3.4. Sensitivity to Control-Point Coordinate Error: Slice 2

Perturbing the control-point world coordinates (N= 10,000 draws per σ) again produces scatter-dominated error, with the mean displacement below 0.7 cm at all noise levels while the scatter grows to 33.7 cm at σ=30 cm (Table 8, averaged across the six evaluation-point pairs). Unlike slice 1, however, the scatter is not linear in control-point noise across the full range. The displacement standard deviation tracks a near-constant 0.76σ up to σ=15 cm, but rises faster above this, reaching 1.12σ at σ=30 cm. This departure coincides with RANSAC beginning to reject one control point as an outlier in a small but growing fraction of draws (its inlier mask, averaged over draws, falls to 0.99 at σ=22.5 and 30 cm), indicating that for this more obliquely viewed slice, the linear regime holds only up to roughly σ=15 cm.

Within the linear regime (σ≤15 cm), the relative scatter is comparable to slice 1, rising from 1.3% at σ=5 cm to 3.8% at σ=15 cm. At the per-point level, the positional scatter slope ranges from 0.77 at e4 to 1.16 at e2 (Table 9), a different spatial ordering from slice 1, reflecting the different geometry of this sensor.

Unlike slice 1, the per-point scatter is not isotropic for all points. Three of the four evaluation points remain close to isotropic (error-ellipse axis ratio ≤1.17), but e2 becomes increasingly elongated as noise grows, reaching an axis ratio of 2.4 at σ=30 cm. This single anisotropic point coincides with the breakdown of the linear regime, and reflects the occasional rejection of a control point at large σ rather than a stable directional effect.

### 3.5. Comparison Between Slices

The two slices agree in the noise range relevant to deployment and diverge beyond it. Baseline distance error is about 2.3× larger for slice 2 (−3.99% mean, against −1.70% for slice 1), and its control-point reprojection RMSE is correspondingly larger (7.9 cm versus 4.4 cm). Under perturbation, both slices are scatter-dominated and follow a near-linear scatter–noise relation up to σ=15 cm, with comparable relative scatter at that level (3.2% for slice 1, 3.8% for slice 2).

**Figure 3:**
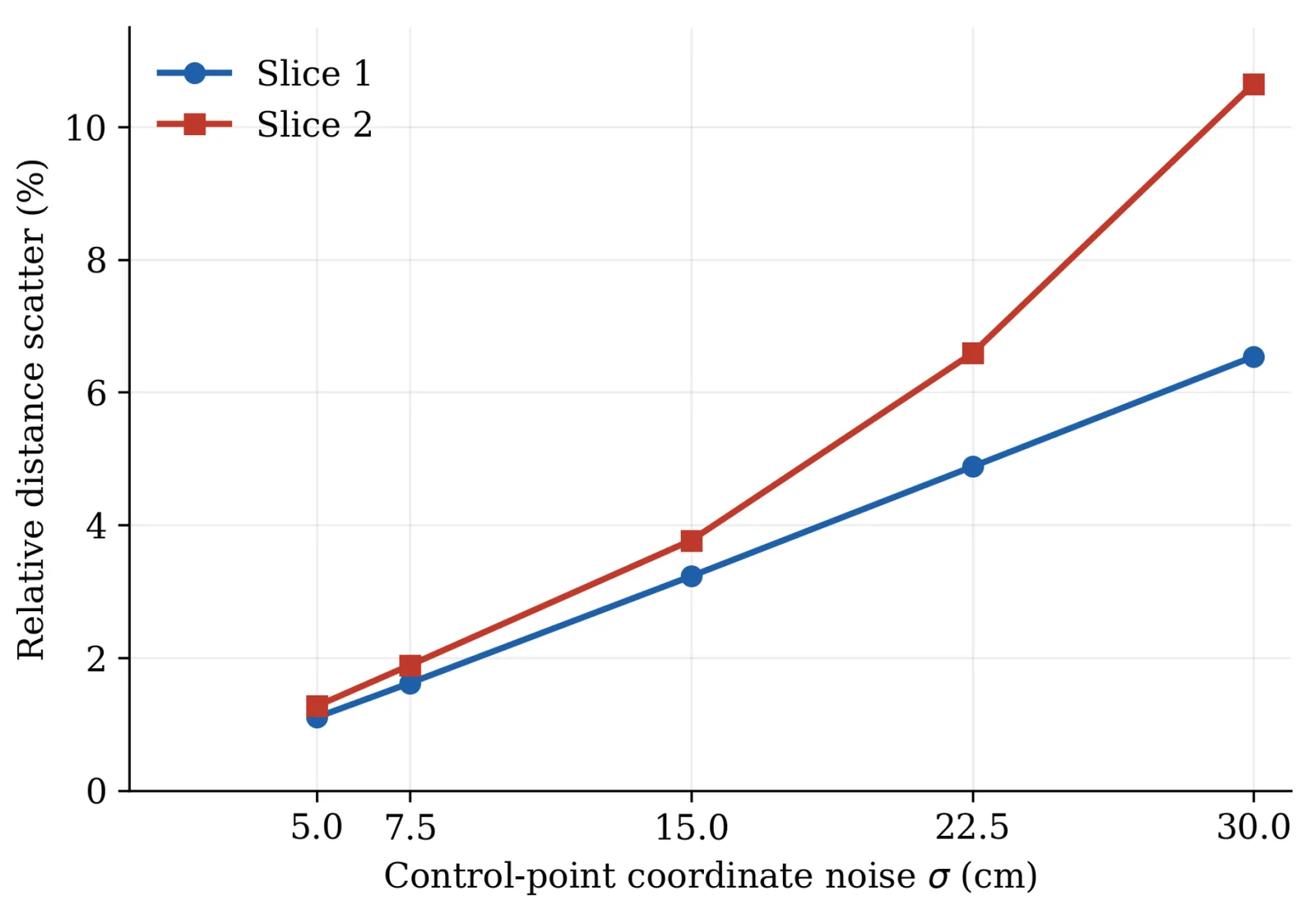
Relative distance scatter vs. control-point coordinate noise σ, both slices.

The slices differ above σ=15 cm (Figure 3). Slice 1 stays linear across the full range, with a scatter slope of about 0.67σ at every noise level, whereas slice 2 departs from linearity, its slope rising from 0.76σ to 1.12σ at σ=30 cm. The per-point scatter is close to isotropic for all four points in slice 1 (error-ellipse axis ratio ≤1.25) and for three of four in slice 2, with one slice 2 point reaching an axis ratio of 2.4 at the largest noise. In both slices, these departures coincide with a rising fraction of draws in which RANSAC rejects a control point at large σ.

In the noise range relevant to satellite-derived or RTK-derived control points (σ≤15 cm), both slices behave alike: the distance error is scatter-dominated and grows approximately linearly with control-point coordinate error, at roughly 0.7 to 0.8 times the per-coordinate noise.

### 3.6. Validation

A separate measurement at the same site and with the same camera checked the pipeline against a reference independent of satellite positioning. On a different day, six points per slice spanning 1.4 to 4.1 m were laid out, with their own control points and their own homographies, and the nine pairwise distances between the six points measured directly with a tape. The projected distances underestimate the tape distances in every pair, by 1.7 to 7.6% in slice 1 and 3.0 to 13.3% in slice 2, reproducing both the consistent underestimation and the ordering of the two slices reported above. Only the site and the camera are shared with the main experiment, so this is an independent replication rather than a recomputation of the baseline; per-pair values are in the data release. The sensitivity result is measured against the unperturbed fit and is unaffected.

An additional experiment evaluated the effect of reducing the number of control points on image-to-ground homography estimation. The main coordinate error experiment was repeated using multiple combinations of 4–7 control points from the available 8, excluding cases with fewer than two enclosed evaluation points to avoid extrapolation. As expected, in both segments, projected distance, absolute error, relative error, and standard deviation increased exponentially as fewer control points were used, as the reduced number of control points provided less compensation for injected coordinate noise. In some extreme cases, the homography fitting process failed.

## 4. Discussion

The results characterise how control-point coordinate error propagates into measured distance for a deployed 180° camera. This section interprets that characterisation: what the bias and scatter mean in practice, how the result compares to existing calibration accuracy figures, what the baseline number does and does not establish, where the two slices agree or disagree, and what this implies for as-deployed pipelines.

**Practical Meaning of Bias and Scatter.** The sensitivity result separates into two parts that behave differently. The bias (signed mean displacement) stays near zero under perturbation, so control-point coordinate error does not push measured distances consistently shorter or longer. The scatter (standard deviation) is the part that grows with the noise, and within the tested range, it is the larger effect. In practice, this means that the error from imperfect control-point coordinates appears as a random spread between repeated measurements, not as a fixed offset that could be calibrated out. For a single measured distance, the scatter sets the expected error: about 0.7 times the per-coordinate noise for slice 1, so a control-point coordinate error of one satellite pixel (≈15 cm) gives roughly 10 cm of distance scatter. Whether this is acceptable depends on the use: for vehicle-level quantities at metre scale it is a few percent, while for finer measurements, it may dominate. This transfers directly to the motivating application, since an inter-vehicle distance is the distance between two vehicle ground-contact points projected through the same homography: the evaluation-point separations of 1.3 to 5.2 m cover typical gaps in queued and urban traffic, and 10 cm of scatter is about 5% of a 2 m gap. Fixed markers stand in for vehicles so that the geometric error is not confounded by contact-point detection, which this paper does not address.

**Comparison with Automatic Calibration Accuracy.** Automatic calibration from scene cues reports a relative-distance accuracy of around two percent [4,5]. Within the noise range relevant here (σ≤15 cm), the relative scatter of the control-point method is of the same order, about three to four percent across the two slices. The two approaches are not directly comparable, since they are evaluated under different protocols and solve different problems: scene-cue calibration infers scale from the scene, while the control-point method fixes scale from surveyed coordinates and has no view redundancy. The comparison is therefore only to place the magnitude of the control-point error on the same scale as the established accuracy figures, not to rank the methods.

**Effect of Camera Calibration.** Calibrating the camera with a target would reduce the residual distortion that contributes to the baseline bias; its effect on the sensitivity can be estimated from first-order error propagation. With known intrinsics, the image-to-road mapping is the camera pose, with d=6 parameters instead of the d=8 of a homography. For *n* points with independent world-coordinate noise σ, the per-coordinate RMS error of the fitted mapping at those points is, to first order, $\sigma \sqrt{d/2n}$ [1], which, for the eight control points used here, is 0.61σ instead of 0.71σ. Calibration would therefore reduce the error propagated from the control-point coordinates by about 13%, but not remove it. The predicted homography value of 0.71σ applies at the control points rather than to distances between held-out points, but it is close to the measured distance scatter of 0.67σ and 0.76σ in the linear regime, so the linear relation found here is the expected first-order behaviour.

**What the Baseline Number Does Not Show.** The baseline distance error is measured against RTK, and both the control points and the evaluation points are surveyed by the same RTK setup. It therefore reflects how well the homography reproduces the survey it was fitted from, together with the residual distortion the planar model does not absorb. It is not an independent accuracy check, because there is no reference of known geometry separate from the RTK survey. The sensitivity result does not depend on this baseline, since it is measured against the unperturbed homography rather than against ground truth, so the absence of an independent reference does not affect the main result.

**Slice-to-Slice Differences.** The two slices start to behave differently at higher noise. Both are scatter-dominated and near-linear up to σ=15 cm, with similar relative scatter. Above this, slice 2 departs from linearity and one of its points becomes anisotropic, while slice 1 stays linear over the full range. The larger baseline error and reprojection residual of slice 2 indicate that its homography fit is less well conditioned, which would explain why it leaves the linear regime earlier under perturbation. Because the two slices come from different sensors, each with its own lens and viewing geometry, this difference is expected; it is not attributable to position within a shared lens, as the panorama has no single optical centre. With only two slices, these observations are indicative rather than general.

**Implications For As-Deployed Measurement Pipelines.** For a deployed camera calibrated from surveyed or orthoimagery-derived control points, the results suggest two practical points. First, since the error is mostly scatter and not bias, effort is better spent on the precision of individual control-point coordinates than on the absolute georeferencing of the whole set: a shift common to all control points is absorbed by the homography and does not change relative distances. Second, the scatter scales approximately linearly with the per-coordinate error in the range tested, so the expected distance error can be estimated directly from the coordinate source: about 0.7 to 0.8 times the per-coordinate standard deviation for the two slices, within the linear regime. The breakdown of the linear regime for the more obliquely viewed slice also suggests that conditioning of the per-slice fit, not only the coordinate accuracy, limits how far this estimate holds.

### Limitations

The scope of this study constrains how its results should be read.

**Limited independent ground truth.** Both the control points and the evaluation points were surveyed with the same RTK setup, so the baseline accuracy reflects fit consistency rather than an independent check against a reference of known geometry. The tape measurement described in Section 3.6 is independent of the survey, but it is a separate run with its own points and homographies, so it shows that the behaviour reproduces rather than validates the reported baseline itself. The sensitivity result is unaffected, since it is measured against the unperturbed homography and does not depend on ground truth.

**Two slices, one camera.** Results come from two adjacent regions of a single 180° camera (AXIS P3807-PVE). The two sensors differ in viewing angle and distortion, so they are treated as two test conditions rather than repeats, but n=2 does not support a general claim across camera models, mounting heights, or lens designs.

**Near-minimal control-point count.** Eight control points were used per slice, close to the minimum needed to fit a homography with some redundancy. The reported sensitivity may be near the upper end of what a more densely controlled setup would show.

**Image-side localisation not varied.** Control and evaluation points were localised in the image using a fixed physical marker, and this image-side error is treated as a constant noise floor rather than measured or varied. The picked pixel is at the same offset from the surveyed point for every point, so this contribution is common to all of them. It is not resolved separately from the model misfit, but the two together are the control-point reprojection residual of 4.4 and 7.9 cm, comparable to the smallest coordinate error swept and well below the 15 cm operating point, so it is not the limiting term.

**Isotropic Gaussian noise model.** Control-point coordinate error is simulated as zero-mean, isotropic Gaussian noise added independently per point. Real error sources (orthoimagery pixel quantisation) are not necessarily isotropic or Gaussian, and may be spatially correlated across nearby points rather than independent. Correlation and quantisation would both reduce the reported scatter: the fully correlated limit is a common shift that the homography absorbs and that cancels in relative distances, and an error uniform over one pixel of size *p* has a standard deviation of $p/\sqrt{12}\approx 0.29\;p$ against the one pixel per standard deviation used here. Anisotropy has no fixed sign but, with eight points spread around the working area, enters the mean scatter only at second order. The model used is therefore conservative rather than neutral.

**Planarity assumed, not measured.** The road surface in each slice is assumed planar; RTK vertical precision was not considered reliable enough in this urban setting to check this assumption directly. Any road camber or crown present would add to the baseline error, but is not separated from other error sources here.

**Residual distortion not corrected.** The image is used as delivered by the camera. The on-device stitching corrects barrel distortion, but a single planar homography does not absorb all residual projection distortion, so the baseline error includes a distortion component that a scene-specific correction would not have. The reported numbers should not be read as the best achievable accuracy for this camera.

## 5. Conclusions

This paper asked how much control-point world-coordinate error, of the kind introduced by reading a point from orthoimagery, changes the ground-plane distance measured by a deployed 180° traffic camera fitted with a single planar homography per region. Using Monte Carlo perturbation of RTK-surveyed control points on two slices of an AXIS P3807-PVE, the error in measured distance is scatter-dominated, with negligible systematic bias, and the scatter grows approximately linearly with the per-coordinate noise, at about 0.7 to 0.8 times the coordinate standard deviation within the tested range. The unperturbed baseline shows a systematic underestimation of the evaluation-point distances of 1.7% and 4.0% for the two slices, which the perturbation does not reproduce and which is therefore attributed to the homography fit and residual distortion rather than to coordinate error.

The two slices agree in the noise range relevant to orthoimagery-derived control points (σ≤15 cm) and diverge above it, where the more obliquely viewed slice leaves the linear regime. The results suggest that, for an as-deployed pipeline, the expected distance error can be predicted from how accurately the control-point coordinates are known, and effort is better directed at the precision of those coordinates than at the absolute georeferencing of the whole set, since a shift common to all control points does not change relative distances. These observations come from two slices of one camera and are indicative rather than general; a broader independent reference, more control-point configurations, and additional cameras would be needed to extend them.

Future work should replicate the measurement across camera models and mounting heights, which the first-order argument predicts would change the coefficient but not the linear form of the relation, and validate the baseline against an independent reference of known geometry such as a total station.

## Figures and Tables

**Table 1:** Representative RTK horizontal precision for a single occupied point (66 samples).

	Latitude	Longitude
Sample stdev	4.2 mm	6.9 mm
Mean GST sigma	14.5 mm	15.2 mm

**Table 2:** Per-point position residual, slice 1, unperturbed *H* vs. RTK truth.

Point Set	Residual Magnitude (cm)	RMSE (cm)
Control points (t1–t8)	3.9±2.1	4.4
Evaluation points (e1–e4)	4.3±2.1	—

**Table 3:** Baseline distance error, slice 1, evaluation-point pairs (unperturbed *H* vs. RTK truth), ordered by true distance.

Pair	True Dist. (cm)	Error (cm)	Error (%)
e1–e2	142.1	−1.29	−0.91
e3–e4	234.0	−3.90	−1.67
e2–e3	328.7	−4.23	−1.29
e1–e3	362.1	−6.05	−1.67
e2–e4	427.9	−10.48	−2.45
e1–e4	521.9	−11.67	−2.24
Mean	—	−6.27	−1.70
Std	—	3.69	0.52

**Table 4:** Per-point position residual, slice 2, unperturbed *H* vs. RTK truth.

Point Set	Residual Magnitude (cm)	RMSE (cm)
Control points (t1–t8)	6.6±4.3	7.9
Evaluation points (e1–e4)	9.1±2.0	—

**Table 5:** Baseline distance error, slice 2, evaluation-point pairs (unperturbed *H* vs. RTK truth), ordered by true distance.

Pair	True Dist. (cm)	Error (cm)	Error (%)
e3–e4	130.0	−3.18	−2.44
e1–e2	291.6	−11.31	−3.88
e1–e3	368.8	−16.40	−4.45
e2–e4	370.6	−17.38	−4.69
e2–e3	410.5	−16.52	−4.03
e1–e4	425.1	−19.02	−4.47
Mean	—	−13.97	−3.99
Std	—	5.37	0.75

**Table 6:** Sensitivity of evaluation-point distances to control-point coordinate noise, slice 1, mean over the six pairs (N= 10,000 draws per σ).

σ (cm)	Mean Disp. (cm)	Std (cm)	Std (%)	Std/σ
5	0.23	3.40	1.10	0.68
7.5	0.26	5.04	1.62	0.67
15	0.47	10.03	3.23	0.67
22.5	0.64	15.15	4.88	0.67
30	1.06	20.34	6.54	0.68

**Table 7:** Per-point positional scatter slope, slice 1: positional RMS scatter per unit control-point noise (RMS/σ, mean over σ).

Eval. Point	RMS Scatter/σ
e1	0.71
e2	0.79
e3	0.79
e4	0.89

**Table 8:** Sensitivity of evaluation-point distances to control-point coordinate noise, slice 2, mean over the six pairs (N= 10,000 draws per σ).

σ (cm)	Mean Disp. (cm)	Std (cm)	Std (%)	Std/σ
5	0.65	3.86	1.27	0.77
7.5	0.64	5.73	1.89	0.76
15	0.70	11.44	3.77	0.76
22.5	0.38	20.40	6.59	0.91
30	−0.18	33.73	10.65	1.12

**Table 9:** Per-point positional scatter slope, slice 2: positional RMS scatter per unit control-point noise (RMS/σ, mean over σ).

Eval. Point	RMS Scatter/σ
e1	0.86
e2	1.16
e3	0.84
e4	0.77

## Data Availability

The data and analysis code are openly available at: https://doi.org/10.5281/zenodo.22749074.
